# LSAP: A Machine Learning Method for Leaf-Senescence-Associated Genes Prediction

**DOI:** 10.3390/life12071095

**Published:** 2022-07-21

**Authors:** Zhidong Li, Wei Tang, Xiong You, Xilin Hou

**Affiliations:** 1 State Key Laboratory of Crop Genetics & Germplasm Enhancement, Ministry of Agriculture and Rural Affairs of the P. R. China, College of Horticulture, Nanjing Agricultural University, Nanjing 210095, China; 2018204017@stu.njau.edu.cn; 2 Key Laboratory of Biology and Genetic Improvement of Horticultural Crops (East China), Engineering Research Center of Germplasm Enhancement and Utilization of Horticultural Crops, Ministry of Education of the P. R. China, Nanjing Suman Plasma Engineering Research Institute, Nanjing 210095, China; 3 College of Sciences, Nanjing Agricultural University, Nanjing 210095, China; 2020111007@stu.njau.edu.cn

**Keywords:** leaf senescence, machine learning, artificial intelligence, classification, database

## Abstract

Plant leaves, which convert light energy into chemical energy, serve as a major food source on Earth. The decrease in crop yield and quality is caused by plant leaf premature senescence. It is important to detect senescence-associated genes. In this study, we collected 5853 genes from a leaf senescence database and developed a leaf-senescence-associated genes (SAGs) prediction model using the support vector machine (SVM) and XGBoost algorithms. This is the first computational approach for predicting SAGs with the sequence dataset. The SVM-PCA-Kmer-PC-PseAAC model achieved the best performance (F1score = 0.866, accuracy = 0.862 and receiver operating characteristic = 0.922), and based on this model, we developed a SAGs prediction tool called “SAGs_Anno”. We identified a total of 1,398,277 SAGs from 3,165,746 gene sequences from 83 species, including 12 lower plants and 71 higher plants. Interestingly, leafy species showed a higher percentage of SAGs, while leafless species showed a lower percentage of SAGs. Finally, we constructed the Leaf SAGs Annotation Platform using these available datasets and the SAGs_Anno tool, which helps users to easily predict, download, and search for plant leaf SAGs of all species. Our study will provide rich resources for plant leaf-senescence-associated genes research.

## 1. Introduction

Plant leaves, which convert light energy into chemical energy, are the main organ for photosynthesis and serve as a major food source on Earth [1]. There has been an increasing concern regarding the decrease in crop yield caused by premature senescence [2]. Many advances in the understanding of the molecular mechanisms of leaf senescence have been achieved, revealing that a large number of senescence-associated genes (SAGs) regulate leaf senescence [1,2]. A leaf senescence database (LSD: https://ngdc.cncb.ac.cn/lsd/, accessed on 1 May 2022) was constructed in 2010 to facilitate systematic studies of leaf senescence. The LSD 3.0 database, presented in 2020, integrates a comprehensive collection of 5853 genes and 617 mutants from 68 species, which provides scientists with useful resources for studies of leaf senescence [3].

Currently, senescence-associated genes are found mainly through biological experiments, which are complex, costly, and labor- and time-intensive. To solve this problem, the use of computational and mathematical methods lies among the most promising alternatives, such as intelligent data mining and knowledge discovery. Machine learning (ML), as a part of artificial intelligence, “learns” a model from empirical data using statistical, probabilistic, and optimization methods in order to predict future data [4]. The support vector machine (SVM), one of many ML methods, is a supervised machine learning technique for classification tasks [4]. The XGBoost algorithm, an integrated learning method, has a stronger generalization ability to obtain better modeling effects. ML has been successfully applied to many bioinformatics problems. For example, Bari et al. built an SVM model to predict a new class of cancer-related genes that were neither differentially expressed nor mutated [5].

Identification of leaf-senescence-associated genes through wet-lab experiments requires more time, human resources, and financial resources. No computational method based on SAGs protein sequence data is available, and that motivated us to propose the present computational method to identify the proteins encoded by the leaf-senescence-associated genes. In this study, we present the Senescence-Associated Genes Annotation Tool (SAGs_Anno), a machine learning method to predict senescence-associated genes from protein sequences. The “SAGs_Anno” tool was developed based on the SVM-PCA-Kmer-PC-PseAAC model (F1score = 0.866, ACC = 0.862 and AUC = 0.922). To facilitate the scientific use of “SAGs_Anno”, we developed the Leaf SAGs Annotation Platform (LSAP: http://www.sagsanno.top:8080/LSAP/index.jsp, accessed on 5 June 2022), based on the “SAGs_Anno” tool. We believe that the LSAP database can be a useful platform for the leaf senescence research community.

## 2. Materials and Methods

### 2.1. Collection of Datasets and Preprocessing

The LSD 3.0 database, presented in 2020, integrates a comprehensive collection of 5853 genes and 617 mutants from 68 species, which provides scientists with useful resources for systematical studies of leaf senescence [3]. The positive data were downloaded from LSD 3.0 (https://ngdc.cncb.ac.cn/lsd/, accessed on 5 May 2022) and further compared to the Pfam (http://pfam.xfam.org/, accessed on 5 May 2022) database [6] using Perl scripts. The positive data included 1638 gene families. Negative data, including 16,291 gene families, were downloaded from the Pfam (http://pfam.xfam.org/, accessed on 5 May 2022) database using Python scripts. To clean the data, we removed the records that contained residues B, J, O, U, X and Z. Additionally, we removed sequences that contained less than 50 amino acids. We also removed the redundant sequences using the CD-HIT program [7] with a threshold of 0.7. Eventually, the filtered dataset contained 6377 and 15,278 protein sequences, which were used to build the classification model.

### 2.2. Features Selection

In this study, three kinds of features, including Kmer, parallel correlation pseudo amino acid composition (PC-PseAAC), and auto-cross covariance (ACC) were employed to construct the SAGs_Anno predictor. Pse-in-one 2.0 software [8], implemented in the Pse-in-One 2.0 database (http://bioinformatics.hitsz.edu.cn/Pse-in-One2.0/, accessed on 26 May 2022), was used to extract features. The nac.py script with k-mer = 2 was used to extract Kmer features. The pse.py script, using the parameters lambda = 2, w = 0.05, was used to extract PC-PseAAC features. Additionally, the ACC features were extracted using acc.py script with LAG = 3.

### 2.3. Machine Learning Model Development

The machine learning prediction model contains many parameters. We needed to determine the optimal values of the parameters through training optimization. We used two machine learning algorithms, namely SVM, provided by the auto-sklearn v0.12.7 package, and XGBoost, provided by the xgboost v1.5.2 package.

### 2.4. Performance Evaluation

In this study, we used fivefold cross-validation to evaluate the performance of our model. We used the F1score, ACC, and AUC as indicators to systematically evaluate the performance of the models from different aspects. We used the pROC v1.16.2 package to calculate AUC scores. The ACC, Precision, Sensitivity and F1score were computed as follows:

(1) $$\mathrm{ACC}=\frac{\mathrm{TP}+\mathrm{TN}}{\mathrm{TP}+\mathrm{TN}+\mathrm{FP}+\mathrm{FN}}$$

(2) $$\mathrm{Precision}=\frac{\mathrm{TP}}{\mathrm{TP}+\mathrm{FP}}$$

(3) $$\mathrm{Sensitivity}=\frac{\mathrm{TP}}{\mathrm{TP}+\mathrm{FN}}$$

(4) $$F1\mathrm{score}=\frac{\mathrm{Precision}\;\times \;\mathrm{Sensitivity}}{\mathrm{Precision}\;+\;\mathrm{Sensitivity}}$$

where TP, TN, FP, and FN represent true positive, true negative, false positive, and false negative, respectively.

### 2.5. Large-Scale Prediction of SAGs

The protein sequences of 83 examined plants were downloaded from the Phytozome [9] database (https://phytozome-next.jgi.doe.gov/, accessed on 5 May 2022), NCBI [10] database (https://www.ncbi.nlm.nih.gov/, accessed on 5 May 2022), Gramene [11] database (https://www.gramene.org/, accessed on 5 May 2022), TAIR [12] database (https://www.arabidopsis.org/, accessed on 5 May 2022), Bolbase [13] database (http://ocri-genomics.org/bolbase/, accessed on 5 May 2021), NHCCDB [14] database (http://tbir.njau.edu.cn/NhCCDbHubs, accessed on 5 May 2022), CuGenDB [15] database (http://cucurbitgenomics.org/, accessed on 15 May 2022), SoyBase [16] database (https://soybase.org/, accessed on 5 May 2022), Ginseng Genome [17] Database (http://ginsengdb.snu.ac.kr/index.php, accessed on 5 May 2022), VIGGS [18] (https://viggs.dna.affrc.go.jp/, accessed on 5 May 2022), RadishDB [19] (http://radish.plantbiology.msu.edu, accessed on 5 May 2022), OAK [20] Database (https://www.oakgenome.fr/, accessed on 5 May 2022), LettuceDB [21] (https://ftp.cngb.org/pub/CNSA/data2/CNP0000335/Other/assembly/, accessed on 5 May 2022), BRAD [22] (https://brassicadb.org/, accessed on 5 May 2022), Brassica napus Genome Resources [23] (https://www.genoscope.cns.fr/brassicanapus/, accessed on 5 May 2022), Barbarea vulgaris Database [24] (http://185.45.23.197:5080/Barbarea_data/, accessed on 5 May 2022), and Banana Genome Hub [25] (https://banana-genome-hub.southgreen.fr/, accessed on 5 May 2022), respectively. To clean the data, we removed the records that contained unknown amino acids using Python codes. The Pse-in-One 2.0 [8] tools were used to extract Kmer and PC-PseAAC features. Based on our presented SVM-PCA-Kmer-PC-PseAAC model, we large-scale predicted plant SAGs from 83 plants.

### 2.6. Database Construction

The LASP (http://www.sagsanno.top:8080/LSAP/index.jsp, accessed on 5 June 2022) database was created by integrating a variety of bioinformatics programs on the Linux platform. This system is set up on an Aliyun server and uses Apache Tomcat as a web server. The collected data were processed using Python codes. All datasets were integrated into the MySQL database. Java, HTML5, JavaServer Pages, CSS3, and jQuery were used to transmit query requirements and extract plant SAGs data from the MySQL database to show in the report pages.

## 3. Results

### 3.1. The Results of SVM Performances

A support vector machine, widely used in bioinformatics and computational biology, is a supervised machine learning technique for classification tasks [4]. The filtered dataset contained 6377 and 15,278 protein sequences, and 80% of the dataset was used to build the classification model. In this study, seven kinds of features, including Kmer, PC-PseAAC, AAC, Kmer-PC-PseAAC, Kmer-AAC, PC-PseAAC-AAC, and Kmer-PC-PseAAC-AAC, were employed to build the SAGs_Anno prediction tool using the SVM method. For SVM, we tuned three hyperparameters, including cost, gamma, and kernel, and we optimized them by using a grid search. Then, 20% of the filtered dataset was used to evaluate the prediction model. Table 1 and Table S1 show the performance of the best prediction model, from which we can see that SVM-Kmer-PC-PseAAC achieved the best performance (F1score = 0.851, ACC = 0.854 and AUC = 0.925), followed by the SVM- PC-PseAAC model (F1score = 0.838, ACC = 0.833 and AUC = 0.900).

Principal component analysis (PCA) is very effective method for data dimension reduction and feature extraction. To further improve the SAGs prediction model, we selected four kinds of combined features, including Kmer-PC-PseAAC, Kmer-AAC, PC-PseAAC-AAC, and Kmer-PC-PseAAC-AAC, and used the PCA method to calculate the discriminative weight vectors in the features space. We chose different dimensional combined features to train the prediction model. Figure S1 shows the performance of the best predictive model. The results show that in four kinds of combined features, Kmer-PC-PseAAC, Kmer-AAC, PC-PseAAC-AAC, and Kmer-PC-PseAAC-AAC, the most discriminative dimensional features numbers are 410, 401, 46 and 161, respectively. Considering task complexity and runtime, we only considered the most discriminative dimensional features to further improve the SAGs prediction model. We trained four predictive models using different combinations of features sets. We tuned three hyperparameters, including cost, gamma, and kernel, and we optimized them using a grid search. The specific features for each combination and number of features, as well as the F1score, ACC, and AUC scores, are shown in Table 2 and Table S1, from which we can see that the SVM-PCA-Kmer-PC-PseAAC model achieved the best performance (F1score = 0.866, ACC = 0.862 and AUC = 0.922), which is better than the SVM-Kmer-PC-PseAAC model.

### 3.2. The Results of XGBoost Performances

The XGBoost algorithm is based on an integrated learning method, which is widely used in the bioinformatics field. In this study, we also used the XGBoost algorithm to train the classification model. For seven kinds of features, including Kmer, PC-PseAAC, AAC, Kmer-PC-PseAAC, Kmer-AAC, PC-PseAAC-AAC, and Kmer-PC-PseAAC-AAC, we trained seven predictive models using the XGBoost algorithm. We tuned six hyperparameters, including max_depth, subsample, min_child_weight, colsample_bytree, gamma, and learning_rate, and optimized them by using a grid search. The performances of the seven predictive models are shown in Table 3 and Table S2. The XGBoost-Kmer-PC-PseAAC-AAC model achieved the best performance (F1score = 0.865, ACC = 0.860 and AUC = 0.925).

For four kinds of combined features, we also used the PCA method to calculate the discriminative weight vectors in the features space, and we chose different dimensional combined features to train the prediction model. The results show that within the four kinds of combined features, Kmer-PC-PseAAC, Kmer-AAC, PC-PseAAC-AAC, and Kmer-PC-PseAAC-AAC, the most discriminative dimensional features numbers are 212, 411, 46 and 425, respectively (Figure S2). After dimension was reduced using the PCA method, the prediction model performance did not improve (Table 4 and Table S2).

### 3.3. A Plant SAGs Predict Tool for Users

We built 22 machine learning models based on two types of learning algorithms: SVM and XGBoost (Table 1, Table 2, Table 3 and Table 4). We can see that the SVM-PCA-Kmer-PC-PseAAC model achieved the best performance, followed by the XGBoost-Kmer-PC-PseAAC-AAC model. Based on the SVM-PCA-Kmer-PC-PseAAC computational model, we developed a tool called “SAGs_Anno” (http://www.sagsanno.top:8080/LSAP/DownloadDetail_detail.action?download_fileType=SAGs_Anno, accessed on 5 June 2022) for proteome-wide identification of proteins encoded by the plant leaf-senescence-associated genes. We also provide instructions on how to use this tool. There are three main functions of this tool: New_data_dealing.py, Pre_SAGs.py, and Pre_result_id.py. Using New_data_dealing.py script, users can remove sequences with residues B, J, O, U, X and Z. After removing such sequences, users can extract Kmer and PC-PseAAC features using Pse-in-One 2.0 tools. With the function Pre_SAGs.py, users can predict plant SAGs based on the SVM-PCA-Kmer-PC-PseAAC computational model. Then, users can extract SAGs id using Pre_result_id.py script. In summary, the developed prediction tool will be of great help to researchers working in the field of identifying plant leaf-senescence-associated genes via wet-lab experiments.

### 3.4. Large-Scale Prediction SAGs

We collected the protein sequences dataset of 83 examined species, which contained 12 lower plants and 71 higher plants, from a public database. The higher plants were further divided into 49 eudicots, 18 monocots, and 4 other higher plants. We identified a total of 1,398,277 SAGs from 3,165,746 gene sequences of 83 species (Table S3).

About half of the species belonged to horticultural plants (Table S3), including 10 fruit trees (*A*. *chinensis*, *A*. *comosus*, *C*. *grandis*, *C*. *canephora*, *J*. *regia*, *M*. *acuminata*, *M*. *domestica*, *M*. *nana*, *P*. *dactylifera*, and *V*. *vinifera*), 14 vegetables (*A*. *officinalis*, *B*. *vulgaris*, *B*. *juncea*, *B*. *oleracea*, *B*. *rapa*, *C*. *annuum*, *C*. *arietinum*, *C*. *lanatus*, *C*. *melo*, *C*. *maxima*, *C*. *sativus*, *D*. *carota*, *L*. *sativa*, and *R*. *raphanistrum*), 13 ornamental plants (*A*. *hypochondriacus*, *A*. *coerulea*, *C*. *cardunculus*, *C*. *grandiflora*, *C*. *nankingense*, *H*. *annuus*, *I*. *nil*, *K*. *fedtschenkoi*, *L*. *angustifolius*, *P*. *equestris*, *R*. *chinensis*, *T*. *cacao*, and *T*. *pratense*), and 4 medicinal plants (*L*. *perrieri*, *M*. *polymorpha*, *P*. *ginseng*, and *S*. *polyrhiza*).

The average SAGs number was 16,846.71, and most species (79, 95.18%) had the SAGs with a number larger than 1000 (Table S3). The average SAGs percentage was 41.92%, and only five species (6.02%) had SAGs with a percentage less than 25%, including *Chlorella variabilis*, *Cyanidioschyzon merolae*, *Chlamydomonas reinhardtii*, *Dunaliella salina*, and *Coccomyxa subellipsoidea*, which belonged to lower plants.

### 3.5. Comparative Analysis of SAGs in Plants

More SAGs were detected in higher plants than in lower plants. The average SAGs number in higher plants (19, 343.97) was 9.5 times that of the average SAGs number in lower plants (2036.08), which may be due to whole-genome duplication and whole-genome triplication events that occurred in most higher plants. Among the top 10 species with a higher percentage of SAGs, all species belonged to the higher plants. Interestingly, of these 10 species, all belonged to eudicots plants. This phenomenon suggests that eudicots plants might contain a higher proportion of SAGs than monocot and other higher plants. All 10 species, including *C*. *rubella*, *C*. *grandiflora*, *A*. *thaliana*, *E*. *salsugineum*, *R*. *raphanistrum*, *B*. *stricta*, *A*. *chinensis*, *S*. *parvula*, *B*. *vulgaris*, and *B*. *juncea,* have a common feature: leafiness.

Among the top 10 species with a lower percentage of SAGs, all species belonged to the lower plants (*C*. *variabilis*, *C*. *merolae*, *C*. *reinhardtii*, *D*. *salina*, *C*. *subellipsoidea*, *O*. *lucimarinus*, *M*. *pusilla RCC299*, *M*. *pusilla CCMP1545*, *C*. *crispus*, and *C*. *braunii*). All 10 species have a common feature: leafless. 

Interestingly, leafiness species showed a higher percentage of SAGs and leafless species showed a lower percentage of SAGs. This phenomenon suggests that genes and plant phenotypes have the same evolutionary trend.

### 3.6. Plant Leaf SAGs Database Construction

**Figure 1:**
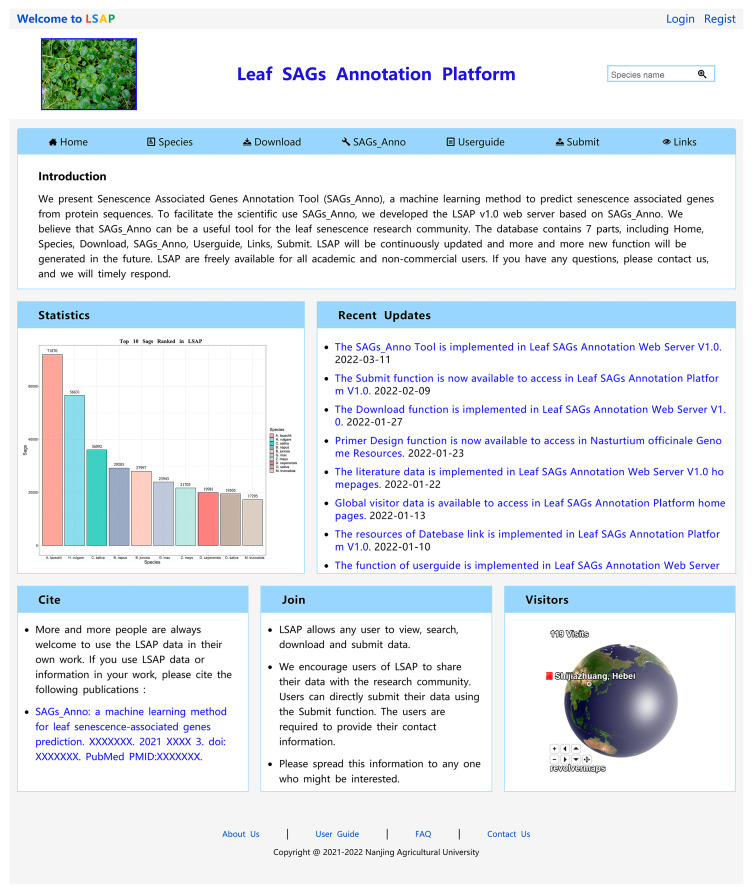
The homepage of the LSAP database.

Using these available datasets, we constructed the Leaf SAGs Annotation Platform (LSAP: http://www.sagsanno.top:8080/LSAP/index.jsp, accessed on 5 June 2022), which helps users to easily predict, download, and search for plant leaf SAGs of all species. The LSAP structure has a user-friendly interface and consists of seven main modules, including Home, Specie, Download, SAGs_Anno, Userguide, Submit and Links (Figure 1).

### 3.7. SAGs_Anno

**Figure 2:**
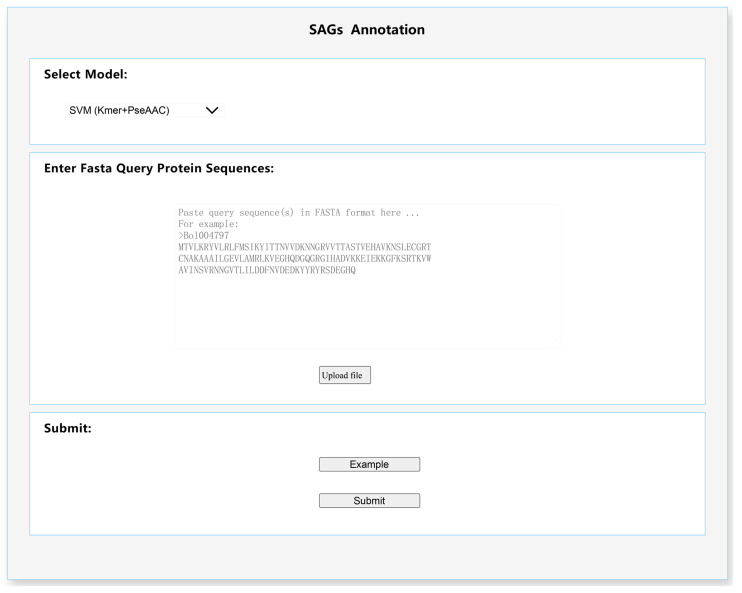
The function of plant leaf SAGs prediction.

Based on the “SAGs_Anno” tool that we developed, we provide an online prediction plant leaf SAGs service using Java, HTML5, and JavaScript. Users only need to supply amino acid sequences in FASTA format, and upon submitting the task. The prediction results can be browsed and downloaded from the results interface (Figure 2).

### 3.8. Browse Examined Species SAGs Dataset

Here, we identified a total of 1,398,277 plant leaf SAGs from 3,165,746 gene sequences of 83 species. The complete SAGs dataset was integrated into the species module. We provided detailed information for each species, including gene identification, coding sequences, protein sequences, and the total number. Scientists can browse and access detailed information about the desired SAGs dataset by clicking the species name.

### 3.9. Download

The Download module has two divisions: the SAGs_Anno and the SAGs dataset. The prediction tool “SAGs_Anno” can be obtained from the SAGs_Anno division. We also provide instructions on how to use this tool in the SAGs_Anno division. The SAGs_Anno division provides the SVM-PCA-Kmer-PC-PseAAC machine learning models. In this division, we also provide positive and negative datasets for the training module. The SAGs module displays a total of 1,398,277 plant leaf SAGs and 83-species SAGs dataset, which contains 12 lower plants and 71 higher plants.

### 3.10. Userguide, Submit, Home, and Links

To help users to access the LSAP database, we provide instructions on how to use this platform. The “Contact Us” function provided at the bottom of every interface contains an e-mail address and phone number to allow users to contact us conveniently and quickly. In the future, we will continue to identify plant leaf SAGs from protein datasets of sequenced species and add them to our LSAP database. To encourage users to submit a new plant leaf SAGs dataset to us, a “Submit” function was embedded in the LSAP. We welcome suggestions from scientists all over the world to further improve our database. We believe that our database will be useful to all researchers.

## 4. Discussion

In this study, we presented a novel computational approach to the recognition of proteins encoded by plant leaf-senescence-associated genes. Compared with biological experiments, this method has the advantages of fast, easy, and inexpensive identification of SAGs. The experimental results showed that our method has a good performance (F1score = 0.866, ACC = 0.862 and AUC = 0.922). The BLAST program [26] has a low recognition rate for non-homology sequences. Compared with the BLAST program, our method has the advantages of high-efficiency and fast identification of SAGs. This is the first computational approach to predicting SAGs with the sequence dataset. Based on the SVM-PCA-Kmer-PC-PseAAC computational model, we presented a tool, “SAGs_Anno”, for the proteome-wide identification of proteins encoded by the plant leaf-senescence-associated genes. We believe that this tool will be of great help to the plant SAGs scientific community. We also predicted large-scale SAGs from protein datasets, which were collected from a public database, and a total of 1,398,277 SAGs were identified from 3,165,746 gene sequences of 83 species. Interestingly, leafy species showed a higher percentage of SAGs and leafless species showed a lower percentage of SAGs. This phenomenon suggests that genes and plant phenotypes have the same evolutionary trend. 

Using these available datasets, we constructed the Leaf SAGs Annotation Platform (LSAP: http://www.sagsanno.top:8080/LSAP/index.jsp, accessed on 5 June 2022), which helps users to easily predict, download, and search plant leaf SAGs of all species. We believe that LSAP will be of great help to all researchers. The uncertainty of a negative dataset is the primary weakness of our method, and we will improve the performance of our method when the LSD database is updated. In the future, more effective features and deep learning techniques, such as convolutional neural networks, recurrent neural networks, and multilayer perceptrons, will be explored to improve our prediction model. In conclusion, this study will serve as a useful resource for future studies on plant leaf-senescence-associated genes.

## Figures and Tables

**Table 1:** The performance of SVM prediction model.

Methods	Number of Feature	F1score	ACC	AUC
SVM-ACC	27	0.811	0.767	0.721
SVM-Kmer	400	0.858	0.857	0.912
SVM-PC-PseAAC	22	0.838	0.834	0.900
SVM-Kmer-ACC	427	0.781	0.787	0.863
SVM-Kmer-PC-PseAAC	422	0.852	0.854	0.925
SVM-ACC-PC-PseAAC	49	0.782	0.789	0.852
SVM-ACC-Kmer-PC-PseAAC	449	0.802	0.807	0.883

**Table 2:** The performance of SVM predictive model using PCA method.

Methods	Number of Feature	F1score	ACC	AUC
SVM-PCA-Kmer-ACC	401	0.816	0.810	0.857
SVM-PCA-Kmer-PC-PseAAC	410	0.866	0.862	0.922
SVM-PCA-ACC-PC-PseAAC	46	0.799	0.797	0.847
SVM-PCA-ACC-Kmer-PC-PseAAC	161	0.822	0.822	0.869

**Table 3:** The performance of XGBoost predictive model.

Methods	Number of Feature	F1score	ACC	AUC
XGBoost-ACC	27	0.790	0.754	0.728
XGBoost-Kmer	400	0.860	0.852	0.916
XGBoost-PC-PseAAC	22	0.840	0.835	0.901
XGBoost-Kmer-ACC	427	0.863	0.854	0.923
XGBoost-Kmer-PC-PseAAC	422	0.860	0.853	0.928
XGBoost-ACC-PC-PseAAC	49	0.850	0.844	0.909
XGBoost-ACC-Kmer-PC-PseAAC	449	0.865	0.860	0.925

**Table 4:** The performance of XGBoost predictive model using PCA method.

Methods	Number of Feature	F1score	ACC	AUC
XGBoost-PCA-Kmer-ACC	411	0. 842	0.832	0.900
XGBoost-PCA-Kmer-PC-PseAAC	212	0.855	0.846	0.919
XGBoost-PCA-ACC-PC-PseAAC	46	0.839	0.829	0.894
XGBoost-PCA-ACC-Kmer-PC-PseAAC	425	0.844	0.832	0.900

## Data Availability

LSAP is freely available at http://www.sagsanno.top:8080/LSAP/index.jsp, accessed on 5 June 2022. The website is optimized for Google Chrome, Internet Explorer, Mozilla Firefox, and Safari.

## Supplementary Materials

The following supporting information can be downloaded at: https://www.mdpi.com/article/10.3390/life12071095/s1, Figure S1: The most discriminative dimensional features number using SVM algorithm; Figure S2: The most discriminative dimensional features number using XGBoost algorithm; Table S1: The hyperparameters of SVM predictive model; Table S2: The hyperparameters of XGBoost predictive model; Table S3: The SAGs data of 83 examined species.
