# Supplementary material for: LSAP: A Machine Learning Method for Leaf-Senescence-Associated Genes Prediction
Source: Life (Basel). 2022 Jul 21;12(7):1095. doi: 10.3390/life12071095 (PMC9316258; doi:10.3390/life12071095)
Supplement: Supplementary file 1 [file life-12-01095-s001.zip › life-1788849-supplementary.pdf]

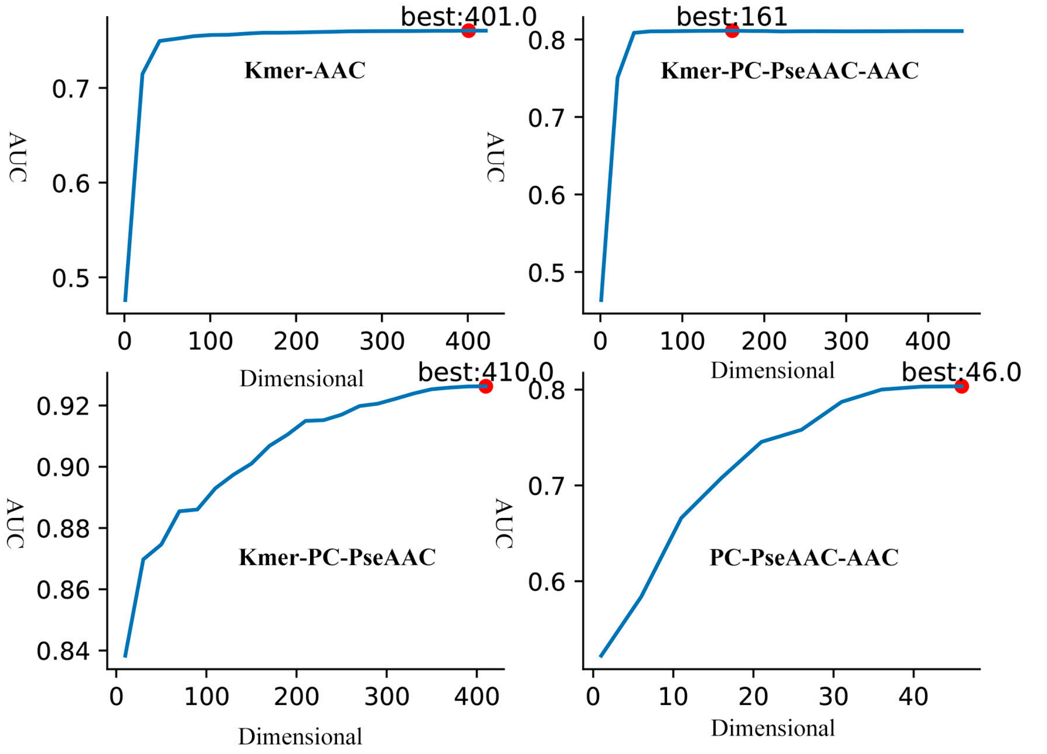

Figure S1: The most discriminative dimensional features number using SVM algorithm.

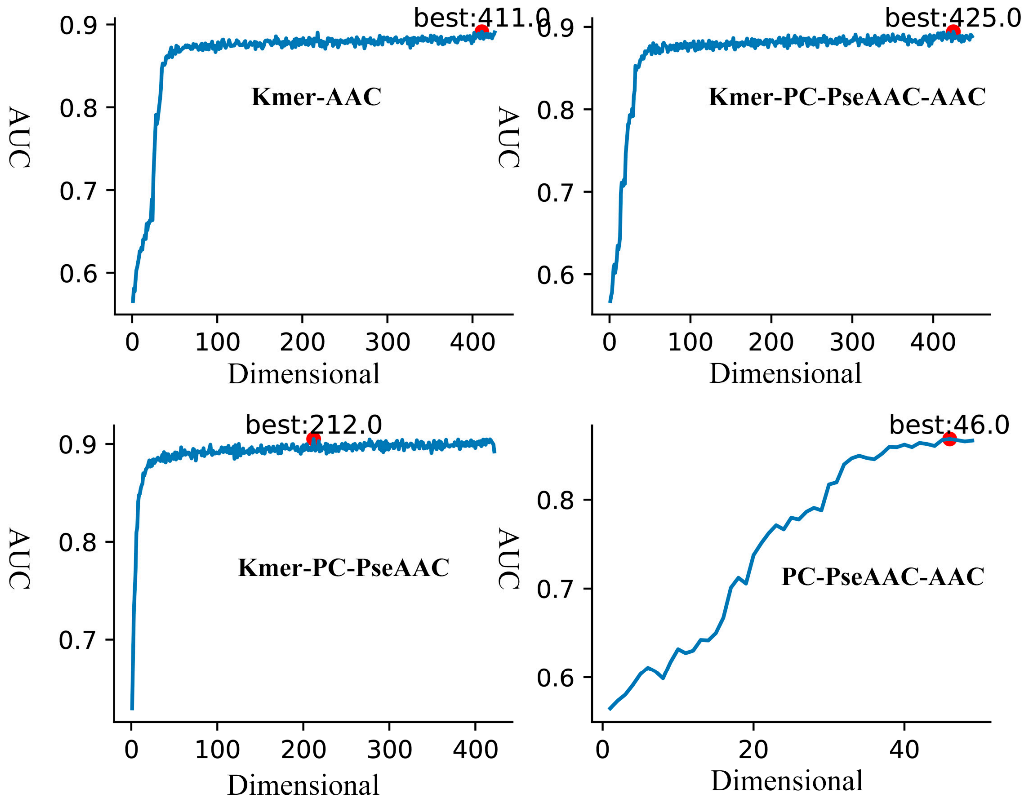

Figure S2: The most discriminative dimensional features number using XGBoost algorithm.

Table S1. The hyperparameters of SVM predictive model.

Methods	Kernel	Gamma	Cost
SVM-ACC	rbf	100	100
SVM-Kmer	rbf	100	100
SVM-PC-PseAAC	rbf	1	1000
SVM-Kmer-ACC	rbf	100	1
SVM-Kmer-PC-PseAAC	rbf	1	100
SVM-ACC-PC-PseAAC	rbf	1000	1
SVM-ACC-Kmer-PC-PseAAC	rbf	100	1
SVM-PCA-Kmer-ACC	rbf	1	100
SVM-PCA-Kmer-PC-PseAAC	rbf	1	1000
SVM-PCA-ACC-PC-PseAAC	rbf	1	1000
SVM-PCA-ACC-Kmer-PC-PseAAC	rbf	1	1000

Table S2. The hyperparameters of XGBoost predictive model.

Methods	Max_depth	Min_child_weight	Gamma	Subsample	Colsample_bytree	Learning_rate
XGBoost-ACC	31	1	0	0.9	0.9	0.05
XGBoost-Kmer	14	6	0	0.8	0.8	0.1
XGBoost-PC-PseAAC	11	1	0	0.8	0.8	0.1
XGBoost-Kmer-ACC	11	1	0	0.8	0.8	0.1
XGBoost-Kmer-PC-PseAAC	11	1	0	1	0.3	0.1
XGBoost-ACC-PC-PseAAC	15	1	0	0.8	0.8	0.1
XGBoost-ACC-Kmer-PC-PseAAC	13	6	0	0.8	0.8	0.1
XGBoost-PCA-Kmer-ACC	15	9	0.3	0.9	0.8	0.1
XGBoost-PCA-Kmer-PC-PseAAC	19	3	0.3	0.9	0.8	0.1
XGBoost-PCA-ACC-PC-PseAAC	19	3	0.1	0.8	0.9	0.1
XGBoost-PCA-ACC-Kmer-PC-PseAAC	23	3	0.1	0.9	0.8	0.1

Table S3. The SAGs data of 83 examined species.

Species	Protein Number	SAGs	Percentage	Classification	Type
Actinidia chinensis	32,876	18,099	0.550523178	Dicots	Fruit trees
Amaranthus hypochondriacus	23,878	9825	0.411466622	Dicots	Ornamental plants
Amborella trichopoda	27,302	10,781	0.394879496	Other higher plant	
Ananas comosus	26,656	12,303	0.461547119	Monocots	Fruit trees
Aquilegia coerulea	43,545	18,803	0.431806178	Dicots	Ornamental plants
Arabidopsis halleri	26,883	14,073	0.523490682	Dicots	
Arabidopsis thaliana	35,381	20,283	0.573273791	Dicots	
Asparagus officinalis	23,120	9637	0.41682526	Monocots	Vegetables
Barbarea vulgaris	25,092	13,743	0.547704448	Dicots	
Beta vulgaris	32,805	15,321	0.467032465	Dicots	Vegetables
Boechera stricta	29,800	16,438	0.551610738	Dicots	
Brachypodium distachyon	52,972	22,500	0.4247527	Monocots	
Brachypodium stacei	35,795	15,613	0.436178237	Monocots	
Brassica juncea	76,349	41,774	0.547145346	Dicots	Vegetables
Brassica napus	100,995	50,468	0.499707906	Dicots	
Brassica nigra	47,925	25,806	0.538466354	Dicots	
Brassica oleracea	35,315	18,446	0.522327623	Dicots	Vegetables
Brassica rapa	48,158	25,776	0.535238174	Dicots	Vegetables
Capsella grandiflora	26,558	15,309	0.576436479	Dicots	Ornamental plants
Capsella rubella	33,542	19,861	0.592123308	Dicots	
Capsicum annuum	34,587	14,538	0.420331338	Dicots	Vegetables
Chara braunii	30,710	7034	0.229045913	Lower plant	
Chenopodium quinoa	34,424	16,390	0.476121311	Dicots	
Chlamydomonas reinhardtii	19,516	1161	0.05948965	Lower plant	
Chlorella variabilis	9776	538	0.055032733	Lower plant	
Chondrus crispus	9622	1681	0.174703804	Lower plant	
Chrysanthemum nankingense	56,870	24,262	0.426622121	Monocots	Ornamental plants
Cicer arietinum	28,022	11,310	0.403611448	Dicots	Vegetables
Citrullus lanatus	22,596	11,624	0.514427332	Dicots	Vegetables
Citrus grandis	42,880	19,257	0.449090485	Dicots	Fruit trees
Coccomyxa subellipsoidea	9629	934	0.09699865	Lower plant	
Coffea canephora	25,571	12,461	0.487309843	Dicots	Fruit trees
Corchorus capsularis	29,353	10,986	0.374271795	Dicots	
Cucumis melo	28,299	12,081	0.426905544	Dicots	Vegetables
Cucumis sativus	23,581	11,750	0.498282516	Dicots	Vegetables
Cucurbita maxima	31,962	17,229	0.539046368	Dicots	Vegetables
Cyanidioschyzon merolae	4972	283	0.056918745	Lower plant	

Cynara cardunculus	25,079	13,426	0.535348299	Dicots	Ornamental plants
Daucus carota	31,861	13,864	0.43514014	Dicots	Vegetables
Dioscorea cayenensis	64,830	23,992	0.370075582	Monocots	
Dunaliella salina	18,173	1703	0.09371045	Lower plant	
Eragrostis curvula	55,173	23,185	0.42022366	Monocots	
Eragrostis tef	40,685	16,197	0.398107411	Dicots	
Eutrema salsugineum	29,280	16,652	0.568715847	Dicots	
Galdieria sulphuraria	7013	1889	0.269356909	Lower plant	
Glycine max	88,406	41,391	0.468192204	Dicots	
Helianthus annuus	52,183	21,428	0.410631815	Dicots	Ornamental plants
Hordeum vulgare	225,723	83,270	0.368903479	Monocots	
Ipomoea nil	41,880	18,582	0.443696275	Dicots	Ornamental plants
Ipomoea triloba	46,904	24,950	0.531937575	Dicots	
Juglans regia	41,077	21,780	0.530223726	Dicots	Fruit trees
Kalanchoe fedtschenkoi	45,044	21,776	0.483438416	Dicots	Ornamental plants
Lactuca sativa	54,434	19,258	0.353786237	Dicots	Vegetables
Leersia perrieri	38,823	18,673	0.480977771	Monocots	Medicinal plants
Lupinus angustifolius	33,073	15,715	0.475161007	Dicots	Ornamental plants
Malus domestica	40,613	19,995	0.492330042	Dicots	Fruit trees
Manihot esculenta	41,393	19,612	0.473799918	Dicots	
Marchantia polymorpha	24,664	7777	0.315317872	Other higher plant	Medicinal plants
Medicago truncatula	14,158	7441	0.525568583	Dicots	
Micromonas pusilla CCMP1545	10,634	1704	0.160240737	Lower plant	
Micromonas pusilla RCC299	10,103	1423	0.140849253	Lower plant	
Musa acuminata	45,586	23,696	0.519808713	Monocots	Fruit trees
Musa nana Lour.	43,041	15,428	0.358448921	Monocots	Fruit trees
Quercus robur	25,631	11,940	0.465842144	Dicots	
Oryza sativa L.	66,326	23,640	0.356421313	Monocots	
Ostreococcus lucimarinus	7769	983	0.126528511	Lower plant	
Panax ginseng	59,117	25,953	0.439010775	Dicots	Medicinal plants
Panicum hallii	43,148	16,069	0.372415871	Monocots	
Phalaenopsis equestris	29,093	15,891	0.546213866	Monocots	Ornamental plants
Phoenix dactylifera	48,266	25,700	0.532465918	Monocots	Fruit trees
Physcomitrella patens	47,945	18,930	0.394827406	Other higher plant	
Raphanus raphanistrum	38,102	21,270	0.558238413	Dicots	Vegetables
Rosa chinensis	47,791	25,075	0.524680379	Dicots	Ornamental plants
Schrenkiella parvula	26,747	14,701	0.549631734	Dicots	

Setaria italica	42,996	17,514	0.407340218	Monocots	
Setaria viridis	38,334	13,612	0.355089477	Monocots	
Sphagnum fallax	45,611	16,081	0.352568459	Other higher plant	
Spirodela polyrhiza	19,482	8682	0.445642131	Monocots	Medicinal plants
Theobroma cacao	44,302	21,502	0.485350549	Dicots	Ornamental plants
Trifolium pratense	35,206	11,729	0.33315344	Dicots	Ornamental plants
Vigna angularis	31,241	13,265	0.424602285	Dicots	
Vitis vinifera	55,415	27,043	0.488008662	Dicots	Fruit trees
Volvox carteri	16,074	5100	0.317282568	Lower plant	
